# Apoptosis signal-regulating kinase 1 (ASK1) inhibition attenuates human airway smooth muscle growth and migration in chronic obstructive pulmonary disease (COPD)

**DOI:** 10.1042/CS20180398

**Published:** 2018-07-31

**Authors:** Mathew S Eapen, Anudeep Kota, Howard Vindin, Kielan D McAlinden, Dia Xenaki, Brian G Oliver, Deepak A Deshpande, Sukhwinder Singh Sohal, Pawan Sharma

**Affiliations:** 1 Department of Medicine, Center for Translational Medicine, Thomas Jefferson University, Philadelphia, PA, USA, 19107; 2 Respiratory Translational Research Group, Department of Laboratory Medicine, College of Health and Medicine, University of Tasmania, Launceston, Tasmania, Australia, 7248; 3 Woolcock Emphysema Centre, Woolcock Institute of Medical Research, Sydney, NSW, Australia, 2037; 4 Medical Sciences Discipline, School of Life Sciences, University of Technology Sydney, Sydney, NSW, Australia, 2007

## Abstract

Increased airway smooth muscle (ASM) mass is observed in chronic obstructive pulmonary disease (COPD) which is correlated with disease severity and negatively impact lung function in these patients. Thus, there is clear unmet clinical need for finding new therapies which can target airway remodeling and disease progression in COPD. Apoptosis signal-regulating kinase 1 (ASK1) is a ubiquitously expressed mitogen-activated protein kinase kinase kinase (MAP3K) activated by various stress stimuli, including reactive oxygen species (ROS), tumor necrosis factor (TNF)-α, and lipopolysaccharide (LPS) and is known to regulate cell proliferation. ASM cells from COPD patients are hyper-proliferative to mitogens *in vitro*. However, the role of ASK1 in ASM growth is not established. Here, we aim to determine the effects of ASK1 inhibition on ASM growth and pro-mitogenic signaling using ASM cells from COPD patients. We found greater expression of ASK1 in ASM-bundles of COPD lung when compared with non-COPD. Pre-treatment of ASM cells with highly selective ASK1 inhibitor, TCASK10 resulted in a dose-dependent reduction in mitogen (FBS, PDGF and EGF; 72 hours)-induced ASM growth as measured by CyQuant assay. Further, molecular targeting of ASK1 using siRNA in ASM cells prevented mitogen-induced cell growth. In addition, to anti-mitogenic potential, ASK1 inhibitor also prevented TGFβ1-induced migration of ASM cells *in vitro*. Immunoblotting revealed that anti-mitogenic effects are mediated by JNK and p38MAP kinase-signaling pathways as evident by reduced phosphorylation of downstream effectors JNK1/2 and p38MAP kinases respectively with no effect on ERK1/2. Collectively, these findings establish the anti-mitogenic effect of ASK1 inhibition and identify a novel pathway that can be targeted to reduce or prevent excessive ASM mass in COPD.

## INTRODUCTION

Chronic obstructive pulmonary disease (COPD) is a chronic inflammatory lung disease that represents one of the most significant global healthcare problems. The worldwide prevalence of COPD according to world health organization (WHO) estimates is over 250 million, and it causes 3 million deaths annually as of 2015, making it third in terms of cause of death and fifth in terms of disease burden worldwide (1, 2). COPD is characterized primarily by slowly progressive airway narrowing with only partial reversibility at most on smoking cessation. Vulnerable smokers develop epithelial squamous metaplasia, mucus hypersecretion, smooth muscle hypertrophy and small airway fibrosis, that causes narrowing and ultimately obliteration, accompanied by “bronchitis” and structural changes throughout the airways (3–6). Up to fifty percent of the patients also go on to suffer from varying degree of emphysematous lung destruction which adds to airflow obstructions and symptoms. In pathological conditions multiple cell types participate in airway remodeling largely through increased extracellular matrix (ECM) deposition and vasculature changes (reviewed in (7, 8)), however our study here will particularly focus on the contribution of airway smooth muscle (ASM) cells in disease manifestation.

An increase in ASM mass, which is commonly associated with airway remodeling in both peripheral and central airways of COPD patients (9, 10). The increase in ASM mass in patients with COPD is believed to generate enhanced contractile force largely due to increased cellular expression of the contractile proteins such as α-smooth muscle actin (αSMA) which cause further narrowing of the airways (11). Increase in smooth muscle thickness leads to increase ECM protein deposition such as collagen-1 (12). Further, size of ASM mass has been shown to correlate with severity of the disease (13, 14). In addition, ASM cells are capable of generating inflammatory mediators, which exacerbate airway obstruction in COPD (15). Despite recent advances in our understanding of the cellular and molecular mechanisms behind COPD (16), the therapeutic options available currently for patients suffering from this illness are fairly limited (17). The events that induces smooth muscle cell growth and migration in COPD are currently unknown and only by understanding the underlying molecular mechanisms that contribute to disease outcome can lead to the development of potential novel therapeutic agents.

The mitogen-activated protein kinase (MAPK) pathway is one of the major signaling pathways that controls a broad range of cellular processes including proliferation, differentiation, development, inflammatory responses and apoptosis (18–20). In the MAPK superfamily, three families have been well characterized, namely CJun N-terminal kinase (JNK), extracellular signal-regulated kinase (ERK) and p38 MAPK (21–23). MAP kinases lie within three-tiered protein kinase cascades. These consist of a series of three enzymes a MAP kinase, a MAPK kinase (MAPKK) and a MAPKK kinase (MAPKKK or MAP3K) (24). While recent work has shown that the inhibition of the MAPK-signaling show therapeutic potential, many of these compounds, such as p38 inhibitors results in toxicity which makes them unsuitable for clinical use (25–27). Thus, a better alternative would be to target upstream kinases that allows selective targeting of specific stress induced signaling activity and eventually minimizing potential toxicity. One such stress-related up-stream kinase of potential interest is apoptosis signal-regulating kinase 1 or ASK1; which in previous reports are shown to have no obvious toxicity induced abnormalities, especially in mouse model of ASK1 deficiency (28). Previous studies have shown that ASK1 is activated by various stresses, including oxidative stress, endoplasmic reticulum (ER) stress and calcium influx and ASK family proteins play key roles in cancer, cardiovascular diseases and neurodegenerative diseases where it regulates cell growth in variety of cell types (29–35) of which oxidative stress is found to be elevated in COPD patients (16). Besides oxidative stress, nitric oxide generated during active inflammatory processes have also been found to increase ASK1-p38 MAPK/JNK cascade *via* induction of activation protein-1 (AP-1) in asthmatic bronchial epithelial cells (36).

ASK1 is a MAPKKK that activates two members of MAPKK, SEK1 and MKK3/MAPKK6, which in turn initiate the JNK and p38 MAPK signaling pathways (30). In this study, we provide evidence of increased expression of ASK-1 in the smooth muscle cells in the airway wall of COPD patients. Further, we demonstrate that both molecular and pharmacological inhibition (37) of ASK1 activity, was sufficient to reduce mitogen-induced ASM growth and migration in cells from patients with COPD. These results indicate that the inhibition of ASK1 help prevent airway remodeling in COPD and highlight ASK1 as a potential therapeutic target for the development of novel small molecule ASK-1 inhibitors for the treatment of COPD.

## MATERIALS AND METHODS

### Acquisition of human lung tissue

Human lung tissues were obtained from surgical resection, explanted lungs and post mortem organ donors with ethical approval from Royal Prince Alfred Hospital (RPAH), Concord Repatriation General Hospital and St Vincent’s Hospital (# HREC14–0045, Sydney) (38). All patients provided consent for their lung tissue to be used for scientific research and in the case of post mortem samples, consent was obtained from the next of kin. Tissue used as non-COPD controls were from non-smoking donors with healthy lungs or from macroscopically normal and isolated regions of lungs from patients with non-small cell carcinoma and free of respiratory or any other systemic diseases.

### Chemicals and reagents

Antibodies against rabbit total and phospho-apoptosis signal-regulating kinase 1 (ASK1), phospho-p42/p44, phospho-p38, phospho-JNK1/2 antibodies were from Cell Signaling Technology (Beverly, MA, USA). IRDye 680 or 800 secondary antibodies were from Rockland (Gilbertsville, PA, USA). CyQUANT cell proliferation assay kit was purchased from Life Technologies (Grand Island, NY, USA). TC ASK 10, an ASK1 inhibitor was purchased from Tocris Biosciences, USA, recombinant TGFβ1 was obtained from R&D Systems, Minneapolis, MN, USA. ON-TARGETplus ASK1 siRNA was purchased from Dharmacon, Mulgrave, Victoria, Australia. Lipofectamine RNAiMAX was purchased from Invitrogen, Australia. All other chemicals were of analytical grade and were obtained from Sigma Aldrich, USA.

### Cell culture

Human ASM cells were obtained from human lung by a method as described previously (39–41). Human ASM bundles were microdissected from approximately 4^th^-6^th^-order bronchii and were initially cultured in growth medium comprised of DMEM:F12 (Invitrogen, Carlsbad, CA, USA) supplemented with 10% fetal bovine serum, 1% antibiotics (Invitrogen, USA.) All the cells tested negative for the presence of mycoplasma before they were set up for experiments and were used between passages 2 and 5. ASM cells were seeded in six-well or 96-well culture plates (BD Biosciences) in growth medium and incubated at 37°C/5% CO_2_. Cells were starved in quiescing medium consisting of DMEM:F12 media supplemented with 1% ITS (Invitrogen, USA), R&D Systems, Minneapolis, MN, USA).

### Cell proliferation assay

Primary human ASM cells obtained from well characterized COPD patients, were plated in either a 96-well plate (CyQUANT assay) and maintained in complete DMEM: F-12 medium supplemented with 10% FBS. After 24 h, cells were switched to growth arresting medium and then treated with growth factors such as 10% FBS, 10 ng/ml PDGF or EGF. Thirty minutes prior to adding growth factors, cells were pretreated with increasing concentration (10 nM to 1 μM) of ASK1 inhibitor TC ASK 10 and compared to equitable DMSO control (vehicle). For growth assay in ASK1 siRNA treated cells, mitogens were used 24 h after ASK1 siRNA transfection. After 72 h treatment with growth factors with vehicle or ASK1 inhibitor or ASK1 siRNA, media was changed to assay buffer containing CyQuant dye and fluorescence intensity measured as per manufacturer’s instructions and as described previously (42).

### Immunoblotting

Human ASM cells obtained from COPD patients were grown to near confluence in 6-well plates and growth arrested for 48 h in serum-free DMEM: F-12 (1%ITS) medium. The cells were then treated with ASK1 inhibitor for 30 min or ASK1 siRNA transfection for 24 h followed by PDGF for 1 h and then cell lysates were collected. For assessing the effect of ASK1 siRNA, non-COPD ASM cells were grown to 70% confluence and then transfected using RNAiMax with 5nM ON-TARGETplus ASK1 siRNA (Dharmacon) and control siRNA for 24, 48 and 72 h. Cell lysates were collected at these three time-points to assess ASK1 knockdown. For collecting protein lysates cells were washed twice with ice-cold buffer (25 mM Tris and 150 mM NaCl, pH 8.0) then solubilized in a 25 mM Tris buffer (pH 8.0) containing 150 mM NaCl, 20 mM NaF, 5 mM EGTA, 1 mM EDTA, 10 mM sodium pyrophosphate, 10 mM p-nitrophenyl phosphate, 1 mM benzamidine, 0.1 M PMSF, and 1% (v/v) Nonidet P-40 (lysis buffer) for 30 min at 4°C. Following scraping, cell lysates were centrifuged at 13,200 g at 4°C for 10 min. Supernatants were collected, then electrophoresed on 10% SDS-polyacrylamide gels, transferred to nitrocellulose membranes, and subsequently probed with the indicated primary antibodies and secondary antibodies conjugated with infrared fluorophores as described previously (42, 43). Immunoblots were visualized and bands quantified using the Odyssey infrared imaging system (LI-COR Biosciences).

### Histopathological analysis

#### Human Lung Tissue Processing and Section Preparation

Dissected lung tissues were fixed in 10% neutral buffered formalin (Sigma-Aldrich, St. Louis, USA). With the use of an automated tissue processor (Excelsior AS Tissue Processor, Thermo Scientific, Waltham, USA), airway samples underwent dehydration process in ascending grades of ethanol and two washes of xylene. Tissue samples were embedded in paraffin for analyses (HistoStar Embedding Workstation, Thermo Scientific, Waltham, USA). Tissue sections cut at 4-micron thickness were prepared using a microtome sectioner (Micron HM325 Rotary Microtome, Thermo Scientific, Waltham, USA) and heated water bath. Following mounting on coated slides (PRO-03; Matsunami, Osaka, Japan), sections were deparaffinised in xylene and rehydrated in graded ethanol prior to staining. Haematoxylin and Eosin (H&E) staining was used to assess the structural integrity, inflammation and the absence or presence of additional pathologies.

#### Immunohistochemistry

Heated antigen epitope retrieval was performed by placing slides in pre-heated (60–90°C) 0.01M citrate buffer, pH 6.0 for 10 minutes and cooling for 30 minutes. After rinsing in water, sections were identified with a hydrophobic Dako Pen (Agilent, Santa Clara, USA) and endogenous peroxidase activity was quenched with incubation in 3% H_2_0_2_ for 10 minutes. Following rinses with water and TRIS buffer (1X tris-buffered saline (TBS)/0.1% tween-20), sections were incubated in DAKO serum-free Protein Block (Agilent, Santa Clara, USA) for 10 minutes. Sections were then washed in tris-buffered saline with tween-20 (TBST) and incubated for 60 minutes with the following diluted primary antibody: rabbit monoclonal anti-ASK1 (1 = 50, Cell Signaling, USA). Bound antibody was elaborated with horse-radish peroxidase (HRP)-labelled EnVision+Rabbit secondary antibody (K4011, Agilent, Santa Clara, USA) incubation for 30 minutes. For each case, the primary antibody was replaced by a species-appropriate isotype-matched immunoglobulin (Rabbit Immunoglobulin Fraction, X0936; Agilent, Santa Clara, USA) for a negative control. The sections were washed firstly in TBST, distilled water, then the antibodies were visualized with the addition of liquid 3,3’-Diaminobenzidine (DAB, Agilent, Santa Clara, USA) and incubated for 10 minutes. The sections were then washed sufficiently in distilled water before nuclei were counter-stained with Mayer’s haematoxylin (Fronine, Riverstone, Australia), blued with ammoniated water (Chem-Supply, Gillman, Australia), and dehydrated and cleared through graded ethanol solutions and xylene. Sections were mounted with Permount (Fisher Scientific, Hampton, USA).

#### Image analysis

Computer-assisted image analysis was performed with a NanoZoomer-SQ Digital slide scanner (Hamamatsu, Hamamatsu City, Japan), Olympus BX51 upright epifluorescence microscope fitted with a DP70 CCD camera (Olympus, Shinjuku, Japan) and Image-J software. Prior to image analysis observer was blinded to subject and diagnosis. After a minimum of 6–7 images per tissue were taken, then four were randomly selected per patient to be quantified. The percentage staining was quantified in the airway wall and in the ASM bundles using Image-J software.

### Wound healing assay

The effect of ASK1 inhibitor on cell migration was measured using wound healing or scratch assay. Briefly, 2×10^5^ cells were seeded in a 6 well plate and incubated for 24 h and then cell monolayer was scraped with a 200 μl pipette tip in a straight line to make a wound. Thereafter ASK1 inhibitor TC ASK 10 at 100 nM was added and incubated for a further 12 h. Images were taken with Olympus BX51 upright epifluorescence microscope fitted with a DP70 CCD camera (Olympus, Shinjuku, Japan) and analyzed using the ImageJ software by measuring the *%* cell-covered area of the wound.

### Data analyses

Values reported for all data represent means ± standard error of means (SEM). For all studies, replicate data from at least 4–7 human donors were obtained. The statistical significance of differences between two means was determined by unpaired t-test or one-way ANOVA with Bonferroni’s multiple comparison test for comparison between treatments or Tukey’s multiple comparison test. Differences were considered to be statistically significant when *p* < 0.05.

## RESULTS

### Expression of ASK1 in the human lung

**Figure 1.**
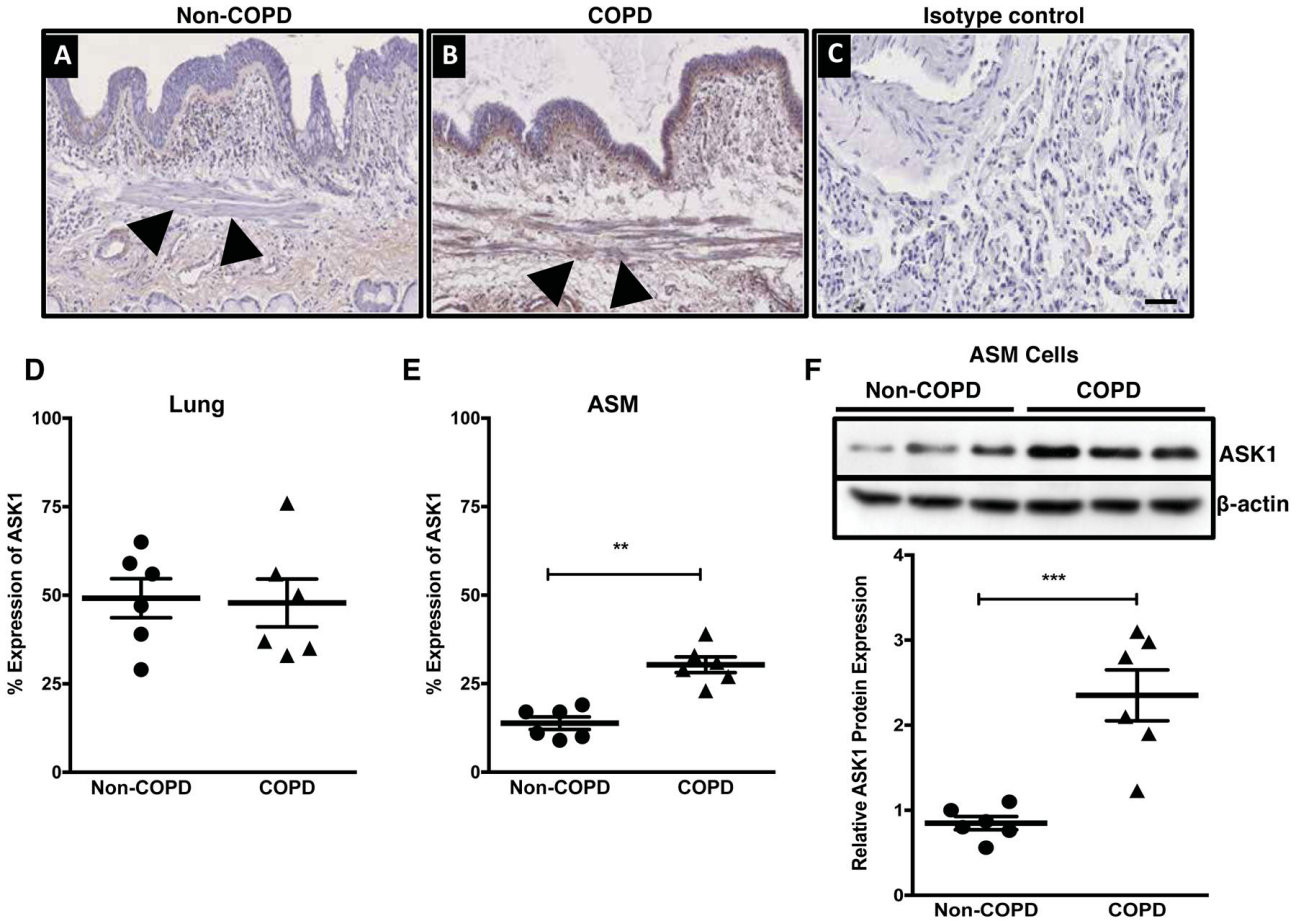
Expression of apoptosis signal-regulating kinase 1 (ASK1) in human lung. IHC analysis of ASK1 in human lung tissue (20x) obtained from healthy (non-COPD, **A**) and diseased lung donors (COPD, **B**), isotype control is shown (**C**). ImageJ analysis of ASK1 expression in the airway wall is shown as % ASK1 expression (**D**) and ASM layer (**E**). Western blot analysis showing expression of ASK1 in human airway smooth muscle cells obtained from non-COPD and COPD donors (**F**). Results are expressed as mean ± SEM. 4–6 patient samples were used in each treatment group. Data were analyzed by unpaired t-test (*p<0.05, **p <0.01, **p <0.001). Arrowheads represents location of airway smooth muscle bundles in the airway. Scale bar, 50 μm.

We first confirmed whether ASK1 is expressed in the human airway wall of controls (non-COPD) and COPD patients (Figure 1A–B). We found that ASK1 was expressed in the airway wall and there were no significant differences between non-COPD and COPD lung (Figure 1D). However, ASK1 expression in the airway smooth muscle (ASM) bundles was significantly higher in COPD patients when compared to controls (Figure 1E). Further confirmation of increased ASK1 expression levels in ASM cells was done for both controls and COPD patients (Figure 1F).

### Pharmacological inhibition of ASK1 attenuates mitogen-induced cell growth

**Figure 2.**
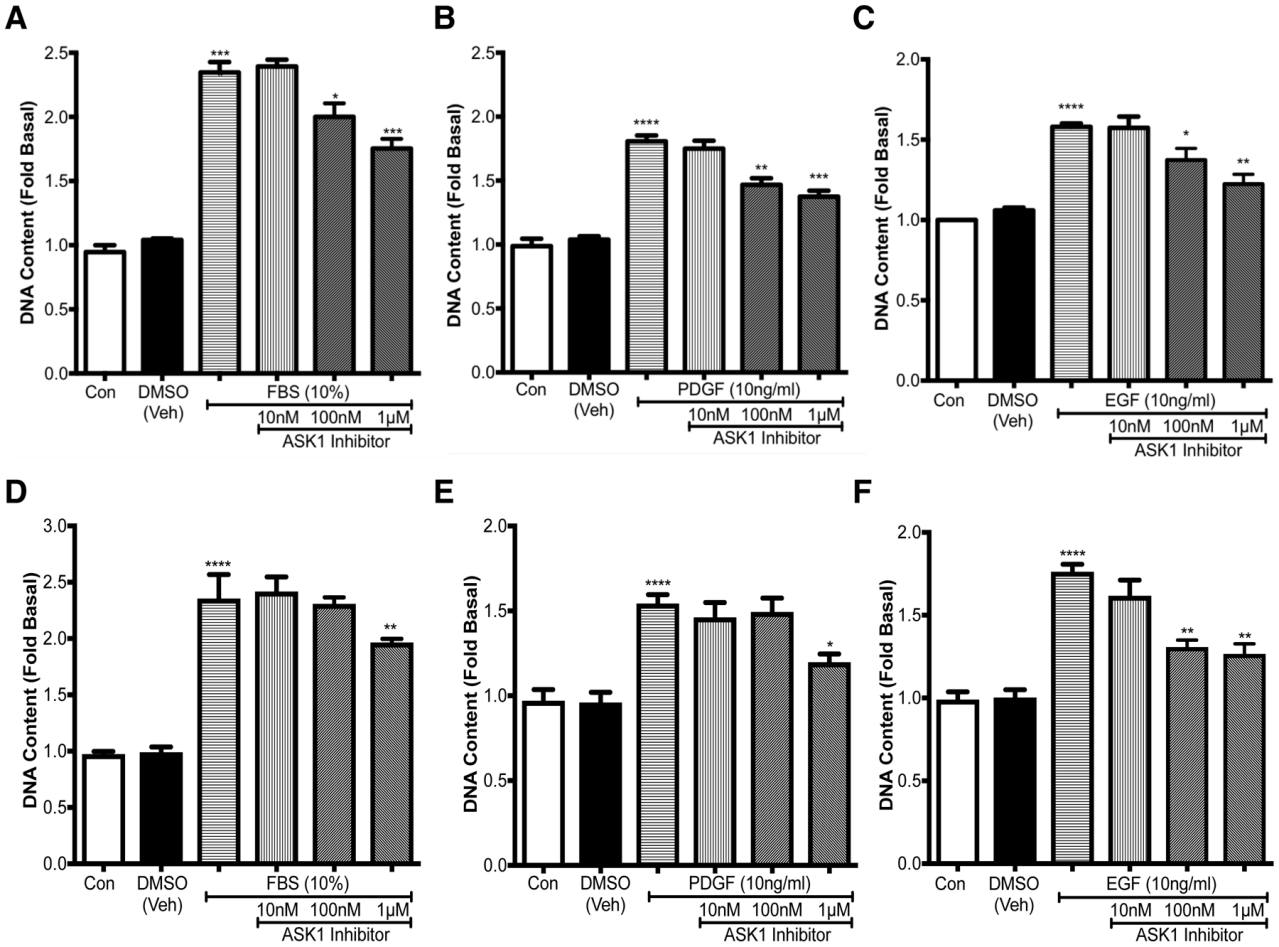
Pharmacological inhibition of ASK1 attenuates mitogen-induced cell growth. ASM cells either from COPD patients (A-C) or non-COPD (D-F) were treated with either 10% FBS (**A, D**). PDGF (**B, E**), or EGF (**C, F**). After 72h fluorescence was measured to assess the increase in DNA content as a measure of cell growth. Results are expressed as mean ± SEM. 4–6 patient samples were used in each treatment group. Data were analyzed by one-way ANOVA followed by Bonferroni’s multiple comparison test (*p<0.05, **p<0.01).

Next, we wanted to test whether pharmacological inhibition using a novel and highly selective ASK1 inhibitor TC ASK 10 affects mitogen-induced ASM growth *in vitro*. Fold change in DNA content was used as a measure of cell growth as this measurement includes both the increase in DNA from both cells that have undergone mitosis and those that have entered the cell cycle and begun the process of division. TC ASK 10 is a potent ASK1 inhibitor (IC50 = 14 nM) and displays high selectivity for ASK1 over other kinases. ASM cells from COPD patients were stimulated with either 10% FBS (Figure 2A), 10 ng/ml PDGF (Figure 2B), or 10 ng/ml EGF (Figure 2C) for 72 h and pre-treated with either DMSO, or TC ASK 10 at increasing concentrations of 10 nM, 100 nM and 1 μM. We found a dose-dependent inhibition of mitogen-induced cell growth with TC ASK 10, with 100 nM and 1 μM being statistically significant (Figure 2 A, B & C). Similarly, we also tested the growth inhibitory capacity of TC ASK 10 in non-COPD cells (Figure 2 D, E & F) where we found similar level of ASM growth with different mitogens as seen in COPD cells with FBS or PDGF or EGF. Interestingly the ability of TC ASK 10 to inhibit mitogen-induced ASM cell growth was reduced as we only obtained statistical differences at the highest concentration (1μM) in FBS (Figure 2D) and PDGF (Figure 2E) treated cells while the inhibition in EGF-treated ASM cells was comparable to COPD cells (Figure 2F). As COPD cells showed greater sensitivity to growth inhibition, for the remainder of this study we only carried out experiments in ASM cells obtained from COPD cells.

### siRNA knockdown of ASK1 attenuates mitogen-induced cell growth

**Figure 3.**
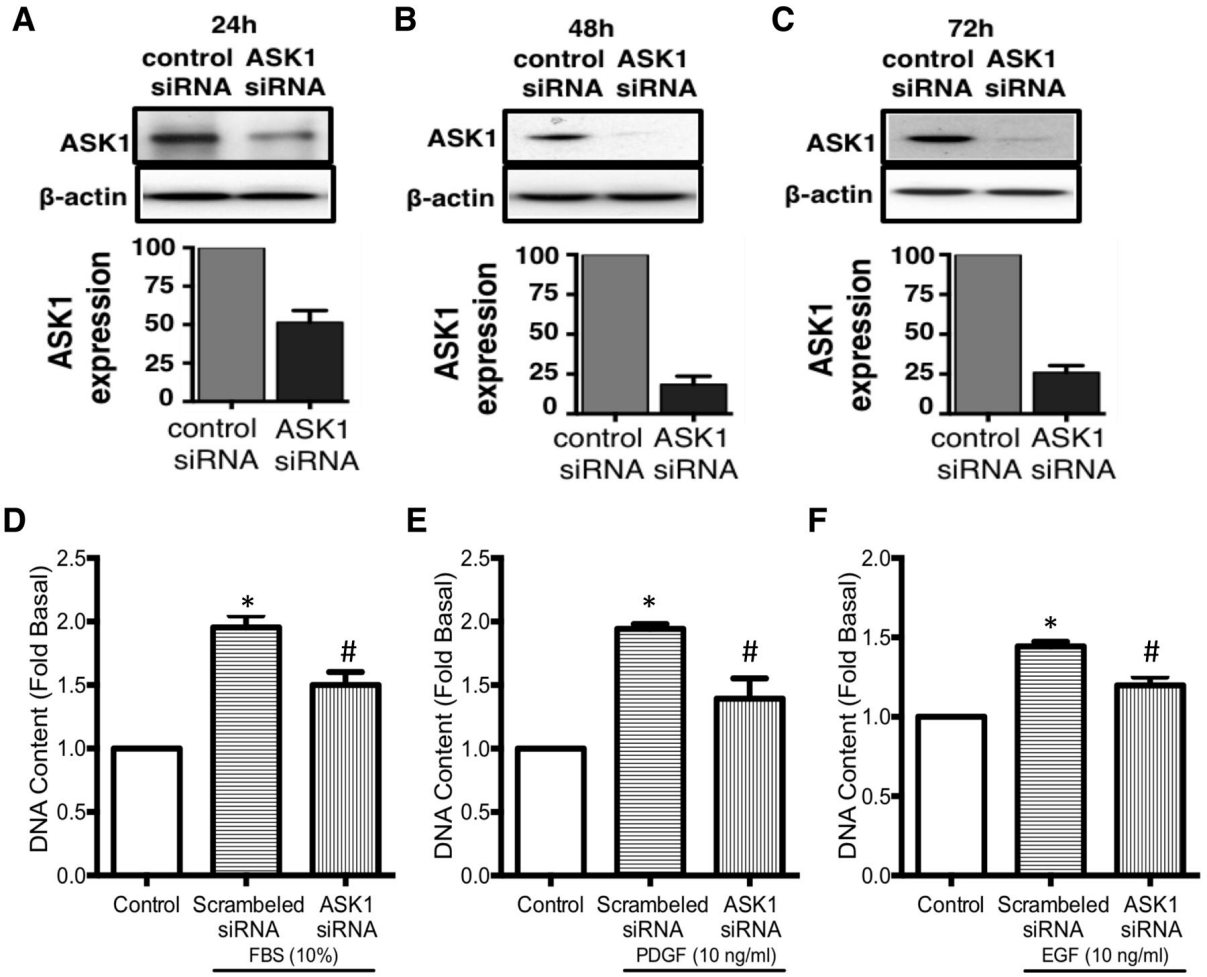
Molecular inhibition of ASK1 in human ASM cells. Western blot analysis showing expression of apoptosis signal kinase 1 (ASK1) in human airway smooth muscle cells obtained from healthy donors upon siRNA knockdown. Protein expression was measured at 24h (**A**), 48h (**B**) and 72h (**C**). Anti-mitogenic effect of ASK1 knockdown was assessed by measuring DNA content in cells treated with either 10% FBS (**D**), 10ng/ml PDGF (**E**) or 10ng/ml EGF (**F**). Results are expressed as mean ± SEM. 4–5 patient samples were used in each treatment group. Data were analyzed by one-way ANOVA followed by Bonferroni’s multiple comparison test; *control *vs* scrambled siRNA; ^#^scrambled siRNA *vs* ASK1 siRNA; *p*<0.05 was considered significant.

We further aimed to validate our findings obtained from pharmacological inhibition of ASK1 by using molecular approach to silence ASK1. We used gene silencing approach to silence ASK1 and assessed mitogen-induced ASM cell growth in COPD. Prior testing this hypothesis we established and validated the successful knockdown of ASK1 gene in non-COPD human ASM cells (Figure 3). Targeted silencing of ASK1 in ASM cells resulted in significant reduction of ASK1 protein at 24, 48 and 72 h (Figure A, B & C) with a peak effect attained at 48 h post transfection (>80% reduction in ASK1 protein, Figure 3B) and this effect being maintained until 72 h (Figure 3C). Next, we checked whether molecular inhibition of ASK1 will have any effect on mitogen-induced ASM cell growth in COPD. This was done by measuring changes in DNA content in ASM cells obtained from patients with COPD and comparing them to controls. We chose 24 h time-point (to get a window where ASK1 levels remains low) following ASK1 siRNA to stimulate cells with either 10% FBS (Figure 3A), 10 ng/ml PDGF (Figure 3B), or 10ng/ml EGF (Figure 3C) for 72 h. Knockdown of ASK1 significantly (*p*<0.05) inhibited mitogen-induced cell growth in all treatment groups (Figure 3A–C) compared to the cell growth in scrambled siRNA transfected cells.

### ASK1 inhibition of mitogen-induced ASM Growth is mediated by JNK and p38 MAP Kinase

**Figure 4.**
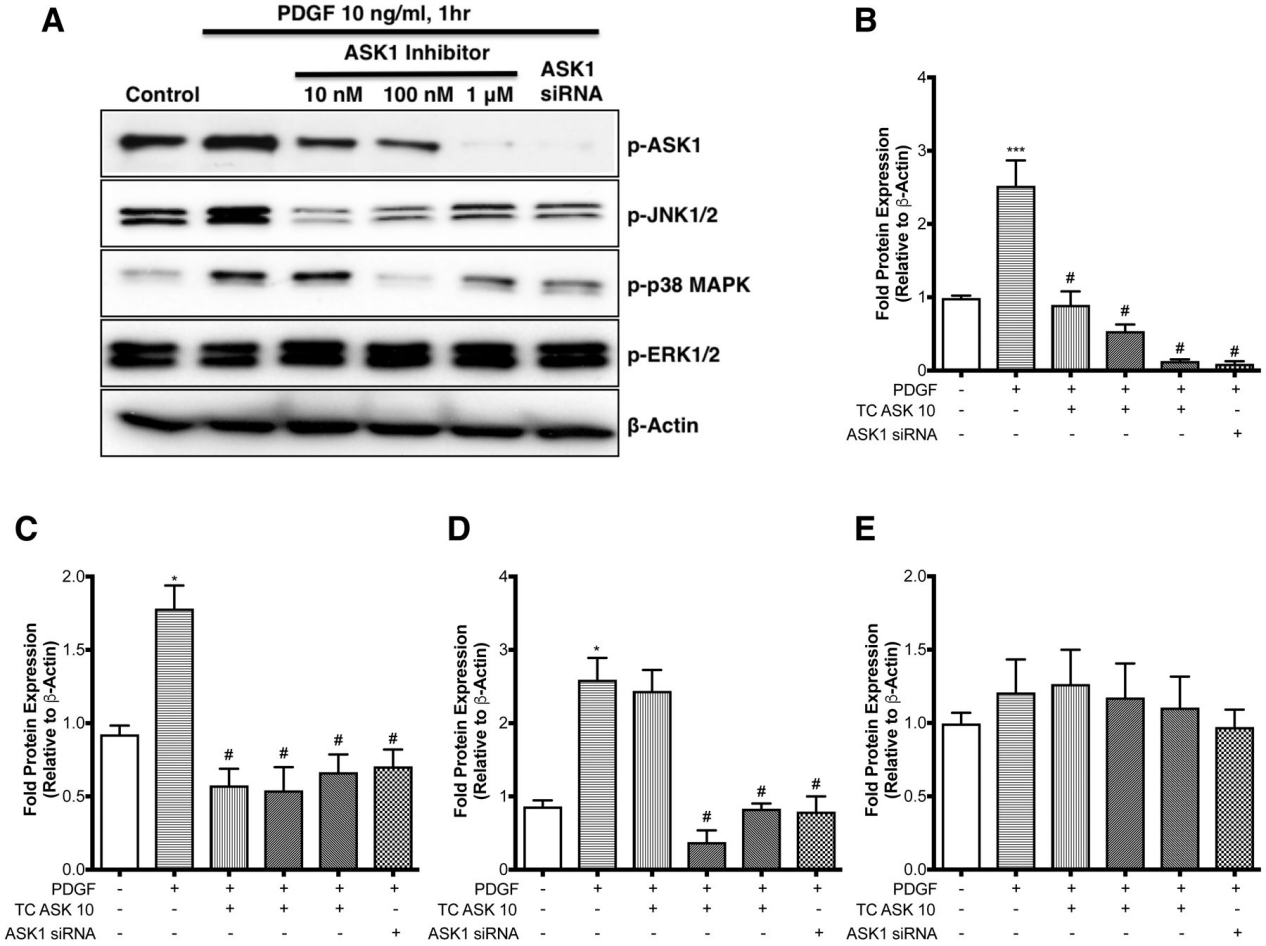
ASK1 inhibition of mitogen-induced ASM growth is mediated by JNK and p38 MAP Kinase. Serum starved human airway smooth muscle cells from COPD patients were treated with PDGF (10 ng/ml, 1h) in presence or absence of either selective ASK1 inhibitor (at indicated concentrations, 30 mins prior to PDGF) or with ASK1 siRNA (24 h time point). Expression of pASK1, pJNK1/2, p-p38MAP Kinase and p-ERK1/2 was determined by Western blot (**A**). Blots are representative of cells taken from at least 4–6 different COPD patients. Mean densitometric analysis is shown for p-ASK1 (**B**), p-JNK1/2 (**C**), p-p38MAP Kinase (**D**) and p-ERK1/2 (**E**). Data were analyzed by one-way ANOVA followed by Bonferroni’s multiple comparison test; *control *vs* PDGF; ^#^PDGF *vs* ASK1 inhibitor (at 10nM or 100 nM or 1μM or ASK1siRNA); *p*<0.05 was considered significant.

To confirm the specificity of TCASK10 in ASM cells we examined the expression of activated MAP kinases JNK, ERK and p38 MAPK following stimulation with PDGF (Figure 4). Stimulated cells were treated with vehicle or TC ASK 10 at concentrations of 10 nM, 100 nM and 1 μM, or transfected with scrambled or ASK1 siRNA. Western blot analysis showed a decrease in levels of phosphorylated JNK and p38 MAPK consistent with previous research demonstrating these pathways are part of the ASK1 signaling cascade (30). There was no change in the expression of phosphorylated ERK at any dose of TC ASK 10, which was expected as ASK1 is not part of the ERK signaling cascade. These results were all consistent with the data obtained from the ASK1 siRNA treatment group. Taken together these findings show that inhibition of ASK1 prevents ASM growth and migration by inhibiting the activation of the downstream signaling cascades JNK and p38 MAPK.

### Inhibition of ASK1 prevents TGFβ-induced cell migration

**Figure 5.**
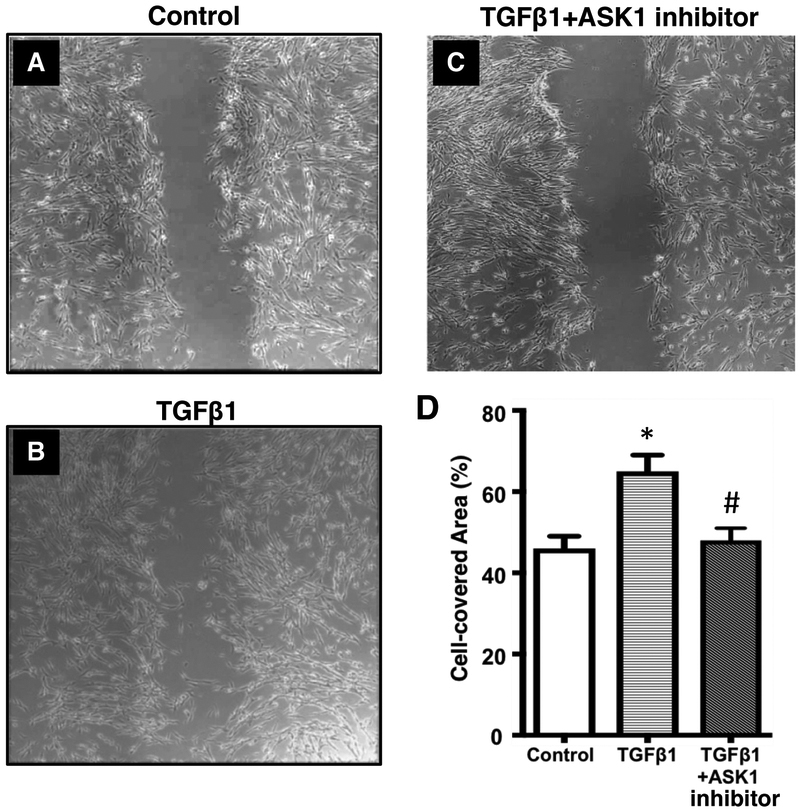
Pharmacological inhibition of ASK1 prevented TGFβl-induced cell migration in COPD. The ASK1 inhibitor TC ASK 10 inhibits cellular migration in airway smooth muscle cells from patients with COPD. Cells were either untreated (control) (A) or treated with either 10ng/ml TGFβl (B) or 10ng/ml TGFβl + 100nM TC ASK 10, an ASK1 inhibitor (C). Migration was assessed by analysing the percentage of wound area covered by cells (**D**). Results are expressed as mean ± SEM. 5–6 patient samples were used in each treatment group. Data were analyzed by one-way ANOVA followed by Bonferroni’s multiple comparison test; *control *vs* TGFβl; ^#^TGFβ1 *vs* TGFβ1+ASK1 inhibitor; *p*<0.05 was considered significant. Scale bar, 100 μm.

To further establish the functional role of ASK1 in ASM function we determined ASM migration. For these studies, we performed a wound healing assay using primary human ASM cell from COPD patients. Cells were treated with TGFβ to stimulate migration either in the presence or absence of TCASK10 (100 nM). Images were taken after 12 h and wound closure was measured as the percentage of the wound area covered by cells (Figure 5 A, B & C). Treatment with TC ASK 10 resulted in significant inhibition of TGFβ-induced wound closure (Figure 5D). These results suggest that ASK1 may be an ideal drug target to prevent ASM migration and attenuate the progressive airway remodeling seen in COPD.

## DISCUSSION

There have been several studies investigating the role of ASK1 in cytokine production, apoptosis and inflammation but very few have focused on its role in cell growth and/or proliferation (25). In the present study, we examined the effect of ASK1 inhibition on cell growth and migration in ASM cells from COPD patients. We found that ASK1 mediates both cell growth and migration in ASM cells. Using both pharmacological and molecular approaches we demonstrate that ASK1 inhibition can lead to reduction in mitogen-induced ASM cell growth. Further our results also showed that ASK1 inhibition effectively reduced TGFβl-dependent ASM cell migration. Excessive ASM cell growth contributes to airway remodeling in COPD and it has been shown that COPD cells are more sensitive to mitogen-induced cell growth, migration and secretion of key pro-inflammatory cytokines. Therefore, effective blocking of ASM cell growth and migration is key to prevent airway remodeling and associated reduced lung function in COPD which ultimately translates into providing long-term clinical benefit.

In the present study we have employed two approaches to target ASK1 in ASM cells i.e. pharmacological and molecular. We have used a novel and highly selective ASK1 inhibitor TC ASK 10 which is highly potent (IC50 = 14 nM) and displays high specificity for ASK1 over other kinases including ASK2 (IC50 = 0.51 μM), MEKK1, TAK1, IKKβ, ERK1, JNK1, p38α, GSK-3β, PKCθ and B-raf (IC50 values are > 10 μM) (37). It is orally bioavailable which is advantageous in COPD as it is a systemic disease. Novel ASK1 inhibitors are being developed and have shown promise in Phase 2 clinical trials for Nonalcoholic Steatohepatitis (NASH), Pulmonary Arterial Hypertension (PAH) and Diabetic Kidney Disease (DKD) (44). The promising results of the novel ASK1 inhibitor in reducing liver fibrosis are highly encouraging as we see similar result with TC ASK 10 where it reduced TGFβl mediated ASM cell migration. These results if translated in COPD will not only reduce mesenchymal cell migration but will also block accumulation and release of extracellular matrix proteins such as collagen and fibronectin in patient with COPD thus providing a net reduction in disease progression which is not yet seen with the existing therapies. This could also have implications for blocking epithelial mesenchymal transition (EMT) in COPD. EMT involves hallmarks such as migration, increased matrix, collagen and fibronectin deposition (45, 46).

The MAPK pathway is a major signaling pathway that controls a myriad of cellular processes (18). MAPK signaling is regulated through the sequential activation of three tiers of protein kinase cascades, MAPKKK, MAPKK, and MAPK (24). ASK1 is ubiquitously expressed MAPK kinase kinase (MAP3K) which is activated by variety of stress stimuli such as reactive oxygen species, TNF-α, LPS, cellular inflammation, calcium. ASK1 plays a key role in cell survival, differentiation and in the development of innate immune response (47, 48). Whilst several studies have investigated the regulation of cellular functions via the JNK and p38 MAPK pathways, relatively few have investigated the effects of upstream MAPKKKs. It has been shown that ASK1 is selectively expressed in many tumor cells and is targeted depletion of ASK1 is beneficial in ventricular remolding (49, 50). Our results in this study have found similar results *in vivo* where we saw an increased expression of ASK1 in the ASM bundles of the airway wall in COPD lung while there were no differences in the other regions of the airway wall in COPD. These results were validated *in vitro* where higher expression of ASK1 was seen in ASM cells obtained from COPD patients when compared to non-COPD controls. There is a limited knowledge on ASK1 in respiratory diseases except that it has been shown to mediate nitric oxide and LTD4-induced AP-1 activation in human bronchial epithelial cells (36, 51). A more recent study demonstrated that ASK1 is required for the induction of experimental asthma in mice with ovalbumin (52). Studies are lacking in determining the role of ASK1 in COPD *per se* and therefore our current study not only uncover potential benefit of ASK1 inhibition in ASM cell growth but also is the first to report the effect of ASK1 inhibition in ASM cells obtained from COPD patients.

Our dual approach using a siRNA and a pharmacological inhibitor to either silence ASK1 gene or block ASK1-mediated signaling is complementary and has demonstrated that ASK1 inhibition using TC ASK 10 resulted in a dose dependent manner reduction in cell growth induced by FBS, PDGF and EGF. We obtained similar results using the ASK1 siRNA, this approach also inhibited mitogen-induced cell growth, a characteristic feature of airway remodeling in COPD. These results show that ASM cell growth in COPD is regulated by ASK1 *in vitro*. We further teased out the downstream signaling pathway that mediate/regulate mitogen-induced cell growth in ASM cells. We found that treatment with TC ASK 10 or with ASK1 siRNA resulted in a reduction in levels of p-JNK and pp38 MAPK, which are directly phosphorylated by ASK1, without effecting levels of p­ERK, whose phosphorylation does not occur through ASK1 (30). These results support previous work that demonstrated inhibition of ASK1 in INS-1 pancreatic β cells as measured by inhibition of downstream phosphorylation of JNK and p38 MAPK (37). One limitation of our study is that we did not test effect of ASK1 inhibition on cigarette smoke-induced ASM cell growth. It has been shown previously by our group that ASM cells from smokers are more sensitive to cigarette smoke stimulation *in vitro* (53). In future we would like to test whether ASK1 inhibition can also modulate cigarette smoke induced cell proliferation and cytokine release *in vitro*.

Further, we also found that the treatment of ASM cells from COPD patients with TC ASK 10 resulted in significant inhibition of TGFβl-stimulated migration in a wound healing assay. These observations provide strong evidence to believe that ASK1 is one of the primary regulatory proteins for this cellular process under pathological conditions. These data indicate that the effects of TC ASK 10 are indeed due to the inhibition of ASK1 and not some other off target effect. While these results suggest that ASK1 may be an ideal candidate for the prevention of ASM cell migration in COPD, the partial inhibition of cell growth may be due to the involvement of other MAPKKKs upstream of JNK and p38 MAPK or due to other signaling events such as ERK1/2 whose role in the regulation of cell proliferation is well known (18, 54). Taken together these results provide additional evidence to support the notion that protein kinases are viable drug targets for the treatment of COPD and this warrants further investigation (55). Further work investigating the contribution of other MAPKKK upstream of JNK and p38 MAPK, as well as the involvement of other signaling pathways may help identify other possible drug targets that selectively inhibit ASM migration for the treatment of COPD and other diseases whose pathogenesis results from deficiencies in normal ASM function such as asthma.

In summary, we found ASK1 inhibition prevented mitogen-induced ASM cell growth and reduced TGFβl-mediated cell migration in COPD. These effects were seen using both molecular and pharmacological approaches using a novel and highly selective ASK1 inhibitor TC ASK 10. These beneficial effects were being mediated by the inhibition of the activity of downstream signaling through p38 MAPK and JNK pathway. Collectively, these findings establish the anti-mitogenic effect of ASK1 inhibition and identify ASK1 as a novel pathway that can be targeted to reduce or prevent excessive ASM mass in COPD.

## Clinical Perspectives

Increased airway smooth muscle (ASM) mass is one of the key drivers of COPD. Current therapies provide only symptomatic relief, they fail to halt disease progression. ASK1 is a MAP3 kinase which affects multiple cellular functions and targeting ASK1 in COPD may provide a novel approach to prevent airway remodeling. In here, we found greater expression of ASK1 in the ASM bundles of COPD lungs, and ASK1 inhibition reduced mitogen-induced ASM cell growth suggesting its role in promoting cell growth and migration that contributes to airway remodeling. These effects are principally mediated by a reduction in p-38MAP-kinase and JNK signaling respectively. Further, we also found that ASK1 inhibition reduced TGFβ1-induced migration of ASM cells in COPD. These findings have greater implication as no current medications effectively reverse structural changes in COPD. We believe the avenue of ASK1 inhibition is an attractive therapeutic target.
